# New Hybrid Nanomaterial Based on Self-Assembly of Cyclodextrins and Cobalt Prussian Blue Analogue Nanocubes

**DOI:** 10.3390/ijms160714594

**Published:** 2015-06-29

**Authors:** Caio L. C. Carvalho, Anna T. B. Silva, Lucyano J. A. Macedo, Roberto A. S. Luz, José M. Moita Neto, Ubirajara P. Rodrigues Filho, Welter Cantanhêde

**Affiliations:** 1 Departamento de Química, Centro de Ciências da Natureza, Universidade Federal do Piauí, Teresina, 64049-550 Piauí, Brazil; E-Mails: caiolenon2011@gmail.com (C.L.C.C.); annathaisebs@gmail.com (A.T.B.S.); lucyanomacedo@gmail.com (L.J.A.M.); jmoita@ufpi.edu.br (J.M.M.N.); 2 Instituto de Química de São Carlos, Universidade de São Paulo, São Carlos, 13563-120 São Paulo, Brazil; E-Mails: luzufabc@gmail.com (R.A.S.L.); uprf@iqsc.usp.br (U.P.R.F.)

**Keywords:** cobalt Prussian blue analogue, β-cyclodextrin, supramolecular, self-assembly

## Abstract

Supramolecular self-assembly has been demonstrated to be a useful approach to developing new functional nanomaterials. In this work, we used a cobalt Prussian blue analogue (PBA, Co_3_[Co(CN)_6_]_2_) compound and a β-cyclodextrin (CD) macrocycle to develop a novel host-guest PBA-CD nanomaterial. The preparation of the functional magnetic material involved the self-assembly of CD molecules onto a PBA surface by a co-precipitation method. According to transmission electronic microscopy results, PBA-CD exhibited a polydisperse structure composed of 3D nanocubes with a mean edge length of 85 nm, which became shorter after CD incorporation. The supramolecular arrangement and structural, crystalline and thermal properties of the hybrid material were studied in detail by vibrational and electronic spectroscopies and X-ray diffraction. The cyclic voltammogram of the hybrid material in a 0.1 mol·L^−1^ NaCl supporting electrolyte exhibited a quasi-reversible redox process, attributed to Co^2+^/Co^3+^ conversion, with an *E*_1/2_ value of 0.46 V (*vs.* SCE), with higher reversibility observed for the system in the presence of CD. The standard rate constants for PBA and PBA-CD were determined to be 0.07 and 0.13 s^−1^, respectively, which suggests that the interaction between the nanocubes and CD at the supramolecular level improves electron transfer. We expect that the properties observed for the hybrid material make it a potential candidate for (bio)sensing designs with a desirable capability for drug delivery.

## 1. Introduction

In recent years, the scientific community has devoted special attention to the development of functional nanomaterials with controlled size and morphology [1,2] due to their combination of small volume and large surface area that allows for the fine-tuning of their physical and chemical properties [2]. To this end, self-assembly has emerged as a potential approach because the concepts of molecular recognition and self-organization are directly related. Using self-assembly, components of various forms and sizes can be suitably chosen and made to interact at the molecular level in different ways to develop functional complex structures [3]. Several materials can be utilized in the self-assembly approach, such as fullerenes [4], carbon nanotubes [5], semiconductor oxides [6] and inorganic complexes [7]. The unique properties of the resulting materials allows them to be used in various devices, such as electronic and electrochemical devices and (bio)sensors and catalysts [8,9,10,11], because the proprieties of nanomaterials differ from those of bulk materials.

Among these materials used in the self-assembly approach, the Prussian blue analogue (PBA) Co_3_[Co(CN)_6_]_2_ is of particular interest. This material is defined as a mixed-valence coordination compound [12,13] containing in its chemical structure Co(II) and Co(III) ions bridged by cyano ligands, as well as one interstitial cobalt center to maintain the electroneutrality of the unit cell of PBA and introduce structural defects [13,14]. This compound arouses interest because it can present interesting magnetic, electrochemical and thermal properties suitable for hydrogen and CO_2_ storage [15,16] and because the compound serves as a promising candidate for use as an anode material in rechargeable lithium ion batteries [17].

The literature reports some studies on the production of PBA nanocrystals for various purposes. For example, Beauvais and Long reported the preparation of microporous ferrimagnetic PBA with an ordering temperature of 38 K by the dehydration of highly crystalline Co_3_[Co(CN)_5_]·8H_2_O, aiming to improve the performance of magnetic separation processes [18]. Cao and coworkers investigated the synthesis of shape-controlled Co_3_[Co(CN)_6_]·for the development of molecule-based magnets [13]. Buchold and Feldmann reported the synthesis of nanoscale, nonagglomerated, and easily dispersible Co_3_[Co(CN)_6_] via the reverse microemulsion technique [19]. Moreover, based on the thermal decomposition of this compound at 520 °C, a monocrystalline, redispersible, magnetic powder of Co_3_O_4_ was synthesized [19].

Other materials that have garnered interest in this field of research are cyclodextrins (CDs), which are defined as macrocyclic oligosaccharides whose natural forms are denoted α-, β- and γ-CDs. These compounds are composed of six, seven, and eight units of glucose, respectively, which are joined together by α-1,4-glycosidic linkages. The molecular arrangement of these compounds into a truncated cone gives rise to a hydrophobic internal cavity and a hydrophilic outer surface [20,21,22,23,24], which enables the use of this molecule for the formation of inclusion complexes with drugs. As such, the CDs have become the target of numerous studies concerning drug delivery systems [25,26,27,28,29,30,31,32]. For example, Silva *et al.* described a study on the design of novel sensing and biosensing systems based on host-guest complexes [33] via the development of supramolecular structures combining Prussian blue nanoparticles, β-CDs, and layer-by-layer method.

In this study, we focused on the development of a new functional nanomaterial formed by the combination of Co_3_[Co(CN)_6_] nanoparticles and β-cyclodextrin with electrochemical and host-guest properties that are of interest in the area of nanomedicine, particularly the formation of inclusion complexes for drug delivery. To this end, the nanomaterial was characterized through UV-visible and FTIR spectroscopies, X-ray diffraction, transmission electron microscopy (TEM) and electrochemical techniques.

## 2. Results

### 2.1. Supramolecular Arrangement

**Figure 1:**
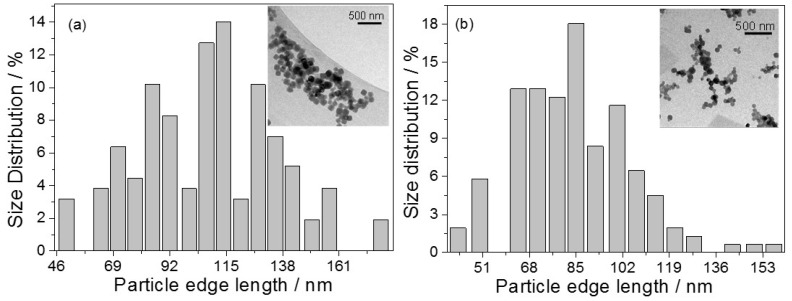
Size distribution histograms for (**a**) PBA and (**b**) PBA-CD nanocubes and corresponding TEM images.

TEM analyses revealed the formation of 3D Co_3_[Co(CN)_6_]_2_ nanocubes (NCs) with a polydisperse distribution. As shown in Figure 1, the histograms of the particle size distribution of the nanocubes show mean edge lengths of 115 (*n* = 157 nanoparticles) and 85 nm (*n* = 155 nanoparticles) for PBA and PBA-CD, respectively. Figure S1 (Supplementary Materials) shows images of well-defined 3D nanocubes.

### 2.2. Spectroscopic Analyses

**Figure 2:**
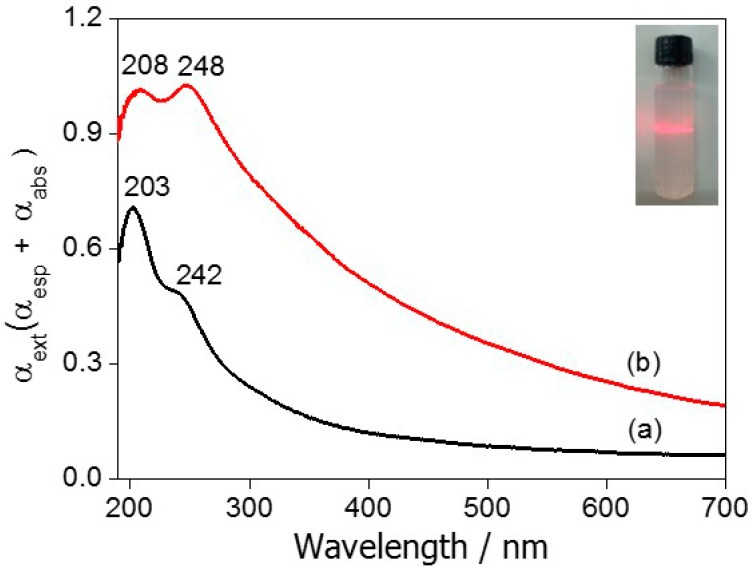
Electronic spectra in the UV-vis region for colloidal suspensions of (a) PBA and (b) PBA-CD. Inset: Tyndall effect for Co_3_[Co(CN)_6_]_2_-CD.

The UV-vis spectra of the materials presented maximum bands (λ_max_) at 203 and 242 nm for PBA and maximum bands at 208 and 248 nm for PBA-CDs [34], as shown in Figure 2. These bands are quite different from those observed for the precursor compounds (Figure S2) [35,36]. The extent of supramolecular organization was estimated based on the ratio between these absorption intensities for the PBA and PBA-CD materials. For the PBA compound (without CD), the *I*_203_/*I*_242_ ratio was 1.47; after the supramolecular arrangement of CD around PBA, the *I*_208_/*I*_248_ ratio decreased to 0.99.

**Figure 3:**
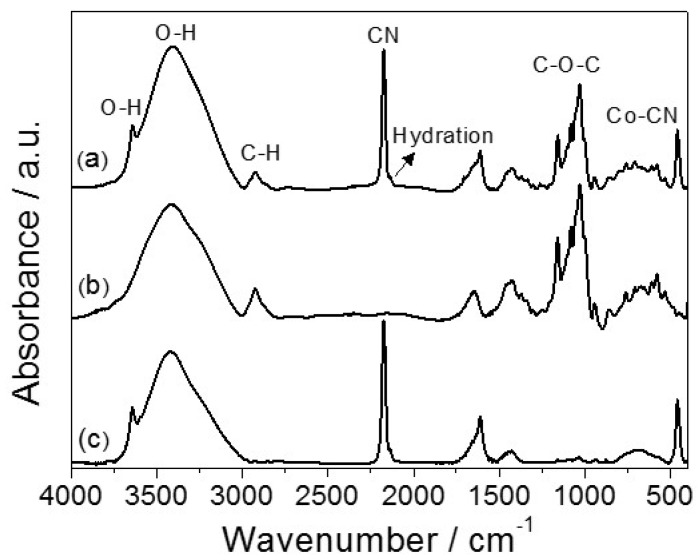
FTIR spectra for (a) PBA-CD (b) CD polymer and (c) PBA.

The FTIR spectra (Figure 3) of the CD, PBA and PBA-CD materials all exhibited a broad characteristic absorption (high intensity) band in the 3750–2900 cm^−1^ region. The peak at 2174 cm^−1^ (high intensity) observed in the PBA samples was attributed to CN stretching in the Co^2+^–CN–Co^3+^ fragment [15]. Other low- and medium-intensity peaks were observed in the spectra and assigned as follows: 2926 cm^−1^ (C–H stretching, CD), 1630 cm^−1^ (H–O–H deformation), from 1155 to 940 cm^−1^ (C–O–C stretching, CD) and 457 cm^−1^ (Co–CN- stretching, PBA) [15,37].

### 2.3. Structure and Crystallinity

The changes in weight percent of the PBA-CD, PBA and CD samples with respect to changes in temperature were investigated by thermogravimetric analysis (TGA). The TGA results illustrated in Figure S3 show that the CD pure sample exhibited three decomposition steps occurring over the temperature ranges of (a) 45–115 °C, assigned to the loss of water (12%) from the CD cavity; (b) 270–355 °C, attributed to the decomposition of the main macrocycle structure, with a weight loss of 66%; and (c) 355–510 °C, assigned to slow carbonization and incineration (22%). For the PBA sample, two events were identified over the temperature ranges of 43–170 and 250–336 °C, corresponding to loss of water (25%) and oxidation of the cyanide (weight loss of 28%), respectively. The isolated PBA-CD product showed two events occurring between 40 and 145 °C and between 230 and 345 °C, with weight losses of approximately of 16% and 59%, respectively, assigned similarly to the events observed for the PBA sample.

**Figure 4:**
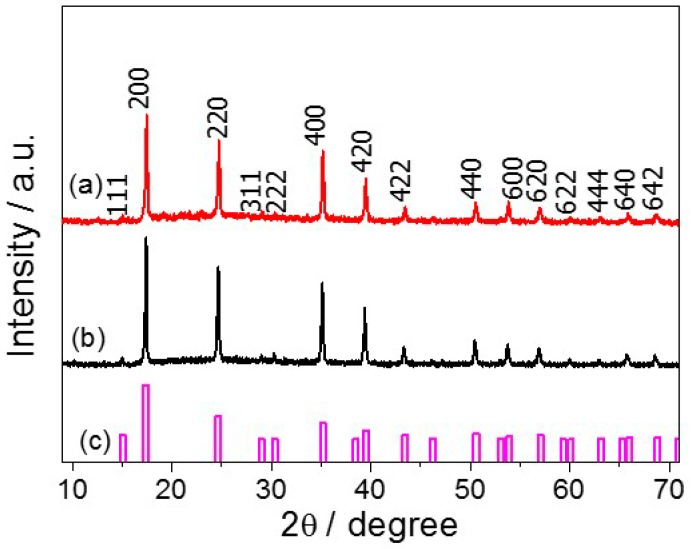
Diffractograms of (a) PBA-CD and (b) PBA nanocubes and (c) crystallographic pattern (JCPDS) of Co_3_[Co(CN)_6_]_2_.

Figure 4 shows the diffractograms of the Co_3_[Co(CN)_6_]_2_ and hybrid materials and the crystallographic pattern of Co_3_[Co(CN)_6_]_2_ (JCPDS No. 77–1161). From 10° to 70°, the PBA-CD material shows 15 main peaks related to PBA [15° (111), 17° (200), 25° (220), 29° (311), 30° (222), 35° (400), 39° (420), 43° (422), 50° (440), 54° (600), 57° (620), 60° (622), 63° (444), 66° (640), and 68° (642)], indicating the presence of the pure face-centered cubic (fcc) phase of Co_3_[Co(CN)_6_]_2_ [13,15].

### 2.4. Electrochemical Study

**Figure 5:**
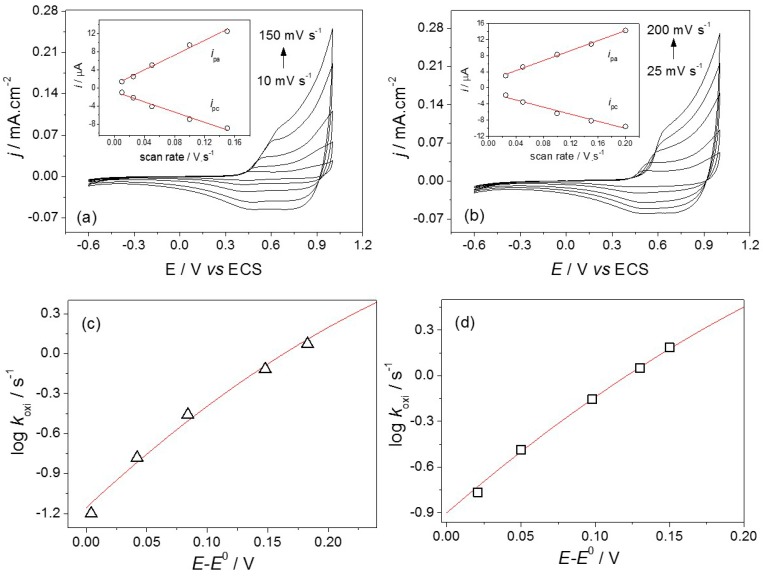
Cyclic voltammograms for (**a**) PBA and (**b**) PBA-CD in 0.1 mol·L^−1^ NaCl recorded at different scan rates. *Inset*: the dependence of the anodic (*i*_pa_) and cathodic (*i*_pc_) current peaks as a function of scan rate; Tafel plots of log *k*_oxi_ *vs*. overpotential (*E*–*E*°) for (**c**) PBA and (**d**) PBA-CD. The solid lines are the fits to Marcus theory, with λ = 0.5 eV for both systems. The anodic intercept (at *E* = *E*°) is –1.15 (*k*° = 0.07 s^−1^) for PBA and −0.9 (*k*° = 0.13 s^−1^) for PBA-CD.

The electrochemical properties of Co_3_[Co(CN)_6_]_2_ and Co_3_[Co(CN)_6_]_2_-CD were investigated by cyclic voltammetry over the range from −0.6 to 1.0 V (*vs.* SCE) in a 0.1 mol·L^−1^ NaCl electrolyte using ITO as the working electrode (electroactive area of 0.16 cm^2^). The voltammogram (Figure 5a) of the Prussian blue analogue showed a quasi-reversible redox couple with a formal potential (*E*°) of 0.46 V and peak-to-peak separation (Δ*E*_p_) of 0.093 V at 0.05 V·s^−1^. The PBA-CD species showed a similar voltammetric profile, but with a Δ*E*_p_ value of 0.061 V (Figure 5b) at the same scan rate.

In both cases, a linear increase in the peak current caused by an increase in the scan rate was observed (inset Figure 5a,b). To evaluate the effect of CD on the electron transfer kinetics of PBA, we estimated the electron transfer rate constants for PBA and PBA-CD as a function of the overpotential (*E*–*E*°), according to the Tafel plots (log *k*_s_ *vs*. overpotential) shown in Figure 5c,d. For each scan rate, the experimental value of *k*_s_ was calculated using equation 1 [38]:

(1) *k*_s_ = *i*_p_/*Q*

where *i*_p_ is the faradaic current and *Q* is the amount of charge involved in the electrochemical reaction, determined by integrating the background-subtracted peaks. The data (symbols) were fitted to theoretical Tafel curves (red lines) predicted by Marcus theory [39,40]. The fit of the anodic branch for both PBA and PBA-CD was excellent, with a reorganization energy (λ) of 0.5 eV. The standard rate constants, *k*°, were estimated (by the intercept at *E* = *E*°) to be 0.07 and 0.13 s^−1^ for PBA and PBA-CD, respectively. 

## 3. Discussion

### 3.1. Characterization of Self-Assembled Supramolecular Nanocubes

**Scheme 1:**
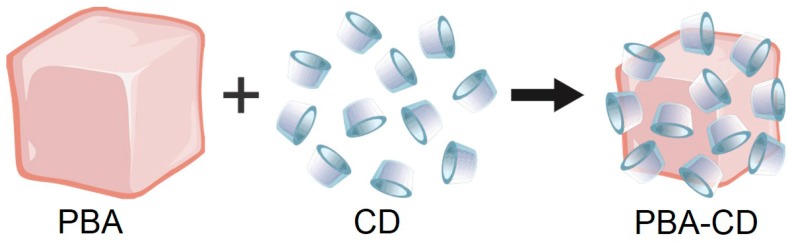
Schematic representation for self-assembly of PBA nanocube and cyclodextrins.

The formation of polydisperse 3D Co_3_[Co(CN)_6_]_2_ nanocubes (with or without CD) was confirmed by TEM images (Figure 1). For Co_3_[Co(CN)_6_]_2_ (without the CD macrocycle), the nanocubes remained closer to one another, which was slightly different from what was observed for the supramolecular arrangement of the PBA-CD system. The nanocubes in each system were not occluded in the CD because the average length of the edges was greater than the size of the macrocycle [41]. Moreover, the mean edge length of the 3D nanocubes decreased in the self-assembled nanoparticles containing CD; it is likely that the PBA nanocubes were capped by the CD macrocycle, thereby affecting nanocube growth. PBA nanocubes have been produced in other studies. For example, using a microemulsion system, Cao and co-workers [13] were able to control both the morphologies and shapes of Co_3_[Co(CN)_6_]_2_ nanocrystals by adjusting the concentration of K_3_[Co(CN)_6_]. However, this method involved the use of various reagents and many processing steps, making the synthesis process laborious and expensive. The Scheme 1 illustrates a schematic of self-assembly of cyclodextrins and cobalt Prussian blue analogue nanocube.

To confirm the TEM observations made in this study, the UV-vis spectra of PBA and PBA-CD were obtained. For the self-assembled PBA-CD material, the bands of PBA showed a slight redshift (bathochromic effect—208 and 248 nm) likely caused by the supramolecular interaction between the 3D PBA nanocubes and the surface-protecting CD polymer, altering the electron transfer energy [33]. It is important to note that the CD species did not show any absorption event from 200 to 900 nm [37]. The discrete effects of the CD polymer on the supramolecular organization of the hybrid material were estimated through the ratio between the absorption intensities of the PBA and PBA-CD materials. The *I*_208_/*I*_248_ ratio for PBA-CD (0.99) was observed to be lower than that for the PBA compound (without CD, 1.47), indicating a local molecular symmetry change in the [Co(CN)_6_] fragment modifying the electronic transition moment for PBA-CD. Indeed, we observed an increase in the baseline for both nanomaterials due to the presence of nano-sized cubes, as the total light absorbance is the sum of the absorbed radiation (σ_abs_) and scattered radiation (σ_sca_) according to the Mie law [42]. The formation of the hybrid nanomaterial was also evidenced by the Tyndall effect, as shown in the inset of Figure 2.

Furthermore, the self-assembly of the CD macrocycle on Co_3_[Co(CN)_6_]_2_ NC surfaces was also investigated by FTIR measurements. For this purpose, spectra for the CD, PBA and PBA-CD materials were obtained and comparatively analyzed. The main stretching and deformation bands presented in the FTIR spectra of the PBA and CD components were also observed in the FTIR spectrum of PBA-CD hybrid. This behavior was confirmed by comparing the theoretical spectrum of the mixture (generated from a combination of the experimental FTIR spectra of PBA and CD) and the experimental spectrum of the hybrid (Figure S4).

The stretching bands that appear near 3645 cm^−1^ for both materials containing PBA were assigned to water molecules bound to metallic cations, which agrees with the above-mentioned observation [43]. In comparing the width at half the peak height of the OH stretching vibration band for PBA (Δν^1/2^ = 375 cm^−1^) and PBA-CD (Δν^1/2^ = 395 cm^−1^), the higher value observed for the hybrid material suggests that CD affected the OH vibrational modes of PBA. The peak at 2174 cm^−1^ (high intensity) observed in the PBA and PBA-CD samples was attributed to CN stretching in the Co^2+^–CN–Co^3+^ fragment, in agreement with previous work on various nanocomposites containing PBA compounds [14,15,43]. According Shriver and Brown [14], the stretching band observed at 2137 cm^−1^ stretching (shoulder, low intensities) is characteristic of the hydration of isolated analogue complexes and PBA-CD. It is worth noting that the self-assembly between CD and PBA at the molecular level is important for the application of the hybrid material in drug delivery studies.

The thermal proprieties observed for the PBA and CD are in good agreement with literature [44,45]. Analysis of the thermogram of the PBA-CD sample revealed that decomposition steps with the same profile proceeded in a manner similar to that observed for the Co_3_[Co(CN)_6_]_2_ sample, but there were slight differences. In the first decomposition step, the water loss was lower for the hybrid material due to the presence of a smaller amount of water. On the other hand, greater weight loss caused by the oxidation of the cyanide occurred after the self-assembly of CD and PBA, likely due to the incorporation of CD onto the surface of the nanocubes. As reported by Yan and coworkers [45], the products of thermal decomposition above 350 °C are species such as Co_3_O_4_ and N_x_O_y_ derivatives for samples annealed in an O_2_ atmosphere. These findings indicate that the thermal stability of the hybrid material is practically the same after the self-assembly of CD.

As previously described, the crystallite size of the nanocubes decreased after CD incorporation; however, the results of the XRD analysis of Co_3_[Co(CN)_6_]_2_ and the hybrid material (Figure 4) agreed with the crystallographic pattern of Co_3_[Co(CN)_6_]_2_ (JCPDS No. 77–1161), which exhibited a well-defined fcc structure. In this case, the presence of amorphous cyclodextrin caused no enhancement in the crystallinity of PBA in either material. Moreover, the thermal stability of PBA-CD was very similar to that observed for isolated PBA, indicating little structural change after the self-assembly of PBA and CD. A closer look at the hybrid diffractogram reveals a peak at 12° assigned to the CD polymer (JCPDS No. 32–1626), as observed in others studies [46].

### 3.2. Electrochemical Characterization and Electron Transfer Study

For the Co_3_[Co(CN)_6_]_2_ and Co_3_[Co(CN)_6_]_2_-CD materials, the quasi-reversible redox couples observed were attributed to the Co^2+^/Co^3+^ couple with the same voltammetric profile, but with a lower Δ*E*_p_ (0.061 V) for the hybrid material, indicating a higher reversibility of the system in the presence of CD (Figure 5b). The linear increase in the peak current caused by the increase in the scan rate indicates that the electrochemical processes are governed by electron transfer at the PBA/electrode interface (inset Figure 5a,b). The standard rate constants for Co_3_[Co(CN)_6_]_2_ and Co_3_[Co(CN)_6_]_2_-CD were estimated to be 0.07 and 0.13 s^−1^ (Figure 5c,d), respectively, which suggests that the interaction between the nanocubes and CD polymer at the supramolecular level improves electron transfer. Although the material system requires further study, this effect may be interesting for the development of sensing and biosensing systems.

## 4. Experimental Section

### 4.1. Chemicals and Materials

All chemicals used were of analytical grade and used without purification. Co(CH_3_COO)_2_·4H_2_O and K_3_[Co(CN)_6_] were commercial products purchased from Acros Organics (Geel, Antwerp, Belgium), whereas the CD polymer and ethanol were acquired from Sigma-Aldrich (St. Louis, MO, USA) and Reagen (Rio de Janeiro, Brazil), respectively. The NaCl compound employed in this study was obtained from Isofar (Duque de Caxias, Rio de Janeiro, Brazil) and used to prepare a 0.1 mol·L^−1^ electrolyte solution. Ultrapure water with a resistivity greater than 18.2 MΩ cm was supplied by a Purelab Option-Q system (Elga Labwater, High Wycombe, Bucks, UK) used for the preparation of all solutions.

### 4.2. Synthesis of Co_3_[Co(CN)_6_]_2_ Nanocubes (Co_3_[Co(CN)_6_]_2_ NCs)

Co_3_[Co(CN)_6_]_2_ NCs were prepared utilizing the method developed Hu *et al.* [15], with some modifications, and used as the experimental control. In a reaction flask, 18.7 mg (7.5 × 10^−5^ mol) of Co(CH_3_COO)_2_·4H_2_O was dissolved in 10 mL of ultrapure water. This precursor solution was slowly added dropwise over 10 mL of 5.0 × 10^−3^ mol·L^−1^·K_3_[Co(CN)_6_] (16.6 mg, 5.0 × 10^−5^ mol) under a nitrogen flow at 25 °C. After this step, a colloidal suspension with slightly pink coloration was observed. The flask containing the product was left for 12 h under constant magnetic stirring until a pink precipitate was observed. The Co_3_[Co(CN)_6_]_2_ powder was separated by centrifugation, washed three times with ethanol to eliminate impurities and finally dried in an oven at 60 °C for 15 min.

### 4.3. Synthesis of Co_3_[Co(CN)_6_]_2_ Nanocubes Decorated with β-Cyclodextrin (Co_3_[Co(CN)_6_]_2_-CD NCs)

Co_3_[Co(CN)_6_]_2_-CD NCs were prepared using a 1:3 stoichiometric ratio between the precursor K_3_[Co(CN)_6_] complex and the β-cyclodextrin matrix. First, a mixture of 10 mL containing 5.0 × 10^−5^ mol (16.6 mg) of K_3_[Co(CN)_6_] and 15 × 10^−5^ mol (170 mg) of β-CD was prepared in concentrations of 5.0 × 10^−3^ and 15 × 10^−3^ mol·L^−1^, respectively. Then, a solution of 7.5 × 10^−3^ mol·L^−1^ Co(CH_3_COO)_2_·4H_2_O was obtained through the dissolution of 18.7 mg (7.5 × 10^−5^ mol) of powder in 10 mL of water pure; the solution was then slowly added dropwise over the initial mixture under a nitrogen flow at 25 °C. After this step, the resulting pink colloidal suspension was left for approximately 12 h under magnetic stirring. Finally, the Co_3_[Co(CN)_6_]_2_-CD powder was separated by centrifugation, washed three times with ethanol and dried at 60 °C for 15 min.

### 4.4. Characterizations

The UV-vis spectra of PBA and PBA-CD colloidal dispersions were obtained with a UV-6100S Allcrom spectrophotometer (Mapada Instruments, Shanghai, China) using a quartz cell with an optical path length of 1.0 cm. A Vertex 70 FTIR spectrometer (Bruker, Billerica, MA, USA) with a measurement range of 4000–400 cm^−1^ was used to record the infrared spectra of the precursor materials and PBAs. The samples were prepared as KBr pellets, and the spectra obtained were normalized with maximum and minimum values corresponding to 1.0 and 0.0, respectively. TEM experiments were performed using a Tecnai 20 transmission electron microscope (FEI, Hillsboro, OR, USA) operated at an accelerating voltage of 200 kV. The copper grids used for imaging (200 mesh, Cu PK/100) were supplied by SPI supplies (West Chester, PA, USA). TEM analysis of the PBA nanocubes was performed after sonicating the suspensions for 5 min. Drops were placed onto a carbon-coated copper grid and dried. Particle counting was performed using the Gatan digital micrograph software package (Pleasanton, CA, USA) after obtaining digital TEM images. A XRD 600 diffractometer (Shimadzu, Nakagyo-Ku, Kyoto, Japan) featuring a Cu-Kα radiation source and operated at a scan rate of 2°/min was utilized to obtain X-ray data (2θ) from 10° to 75° under a continuous scan mode. Thermogravimetric analysis (TGA) was performed using a SDT Q600 V20.9 Build 20 (TA Instruments, New Castle, DE, USA) from 25 to 700 °C with a temperature ramp of 20 °C·min^−1^ in air. An Autolab 128N potentiostat/galvanostat (Metrohm, Kanaalweg, Utrecht, The Netherlands) coupled with a standard three-electrode cell was used to conduct cyclic voltammetry measurements. The saturated calomel electrode, a platinum wire and a bare ITO slide (R_s_ = 70–100 Ω, Delta Technologies Ltd., Auburn Hills, MI, USA) were used as the reference, counter and working electrodes, respectively, with 0.32 mg·mL^−1^ of the as-synthesized nanomaterial dispersed in 0.1 mol·L^−1^ NaCl (pH 7.2) electrolyte; the system was purged with nitrogen flow over 5 min.

## 5. Conclusions

The self-assembly of a CD macrocycle on the surface of Co_3_[Co(CN)_6_]_2_ was successfully performed, yielding small and aggregated 3D nanocubes in the presence of CD, as revealed by TEM. The incorporation of CD also affected the extent of Co^2+^/Co^3+^ conversion, leading to a more reversible voltammetric profile and an increase in the standard rate constants, suggesting that the interaction between the nanocubes and CD polymer at the supramolecular level improves electron transfer. As suggested by UV-vis and FTIR spectra, the CD macrocycle and PBA compound formed a supramolecular structure, causing, in particular, a change in the electronic transition moment for PBA-CD via a bathochromic effect. However, the crystallinity and thermal proprieties of Co_3_[Co(CN)_6_]_2_-CD were not affected by the CD species, as indicated by XRD and TGA. The developed Co_3_[Co(CN)_6_]_2_-CD hybrid material is promising for host-guest studies concerning the design of (bio)sensing systems.

## Supplementary Materials

Supplementary materials can be found at http://www.mdpi.com/1422-0067/16/07/14594/s1.
