# Supplementary material for: New Hybrid Nanomaterial Based on Self-Assembly of Cyclodextrins and Cobalt Prussian Blue Analogue Nanocubes
Source: Int J Mol Sci. 2015 Jun 29;16(7):14594–607. doi: 10.3390/ijms160714594 (PMC4519860; doi:10.3390/ijms160714594)
Supplement: Supplementary file 1 [file ijms-16-14594-s001.pdf]

Supplementary Information

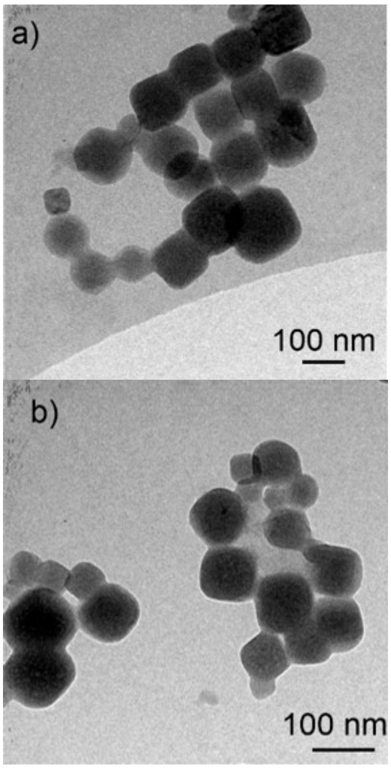

Figure S1. TEM images with scale bars in each micrograph measure 100 nm for (**a**) PAB nanocubes and (**b**) PAB-CD nanocubes.

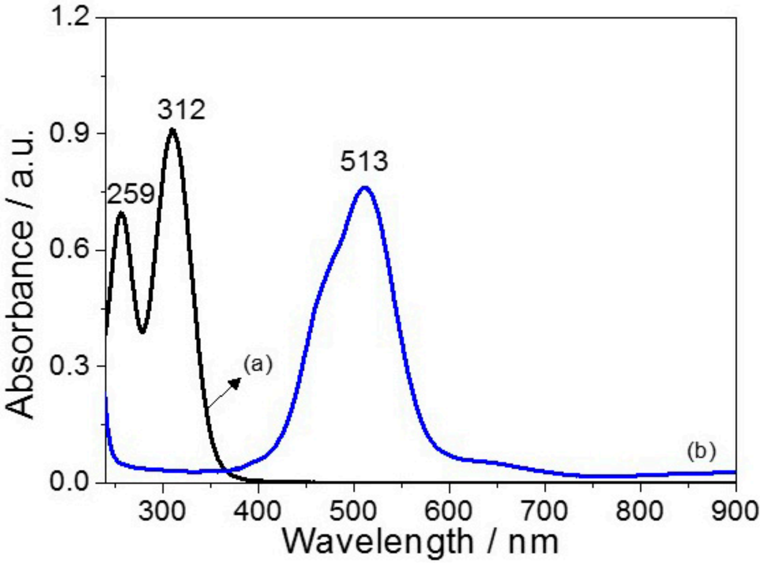

Figure S2. UV-Vis spectra for: (a) 3 mmol·L^{-1} $K_3[Co(CN)_6]$ and (b) 0.1 mol·L^{-1} $Co(CH_3COO)_2 \cdot 4H_2O$ solutions.

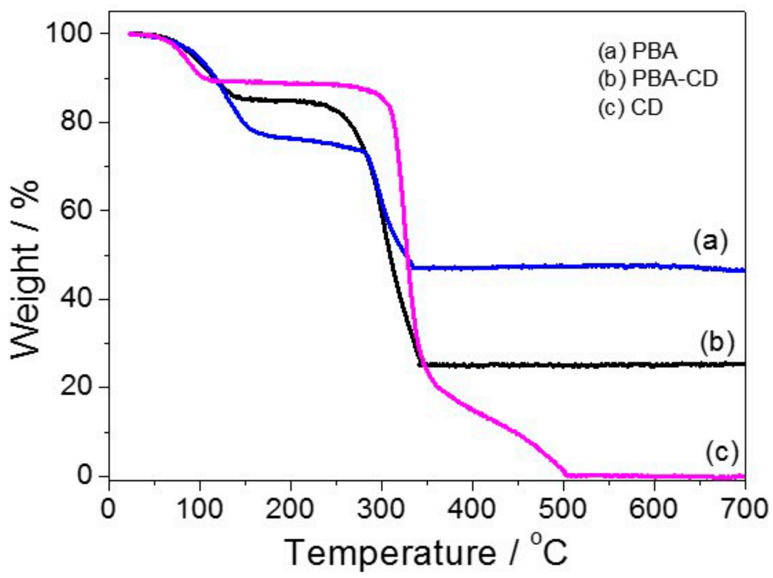

Figure S3. The TGA scans for (a) PBA NCs; (b) PAB-CD and (c) CD.

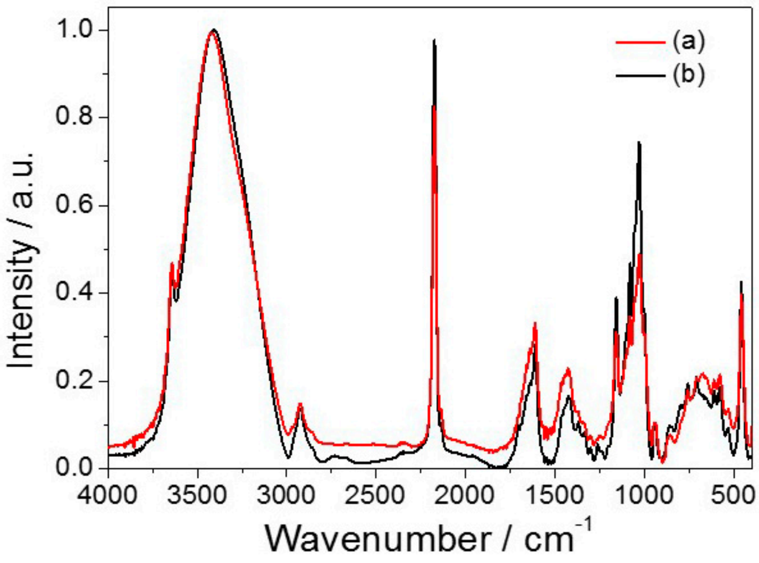

Figure S4. FTIR spectra for (a) theoretical spectrum of the mixture (generated from combination of the experimental FTIR spectra of PBA and CD) and (b) experimental spectrum of hybrid.
